# Detection of CpG Methylation in G-Quadruplex Forming Sequences Using G-Quadruplex Ligands

**DOI:** 10.3390/ijms222313159

**Published:** 2021-12-06

**Authors:** Hijiri Hasegawa, Ikkei Sasaki, Kaori Tsukakoshi, Yue Ma, Kazuo Nagasawa, Shusuke Numata, Yuuki Inoue, Yeji Kim, Kazunori Ikebukuro

**Affiliations:** 1 LG Japan Lab Inc., 4-13-14 Higashi Shinagawa, Shinagawa-ku, Tokyo 140-0002, Japan; hijiri.hasegawa@lgjlab.com (H.H.); yuuki.inoue@lgjlab.com (Y.I.); yeji2.kim@lgjlab.com (Y.K.); 2 Department of Biotechnology and Life Science, Tokyo University of Agriculture and Technology, 2-24-16 Naka-cho, Koganei, Tokyo 184-8588, Japan; ikkei0922@gmail.com (I.S.); k-tsuka@cc.tuat.ac.jp (K.T.); knaga@cc.tuat.ac.jp (K.N.); 3 Institute of Global Innovation Research, Tokyo University of Agriculture and Technology, 2-24-16 Naka-cho, Koganei, Tokyo 184-8588, Japan; yue-ma@m2.tuat.ac.jp; 4 Department of Psychiatry, Graduate School of Biomedical Sciences, Tokushima University, 3-8-15 Kuramoto-cho, Tokushima 770-8503, Japan; shu-numata@tokushima-u.ac.jp

**Keywords:** DNA methylation, G-quadruplex, G4 ligand, biosensor

## Abstract

Genomic DNA methylation is involved in many diseases and is expected to be a specific biomarker for even the pre-symptomatic diagnosis of many diseases. Thus, a rapid and inexpensive detection method is required for disease diagnosis. We have previously reported that cytosine methylation in G-quadruplex (G4)-forming oligonucleotides develops different G4 topologies. In this study, we developed a method for detecting CpG methylation in G4-forming oligonucleotides based on the structural differences between methylated and unmethylated G4 DNAs. The differences in G4 topologies due to CpG methylation can be discriminated by G4 ligands. We performed a binding assay between methylated or unmethylated G4 DNAs and G4 ligands. The binding abilities of fluorescent G4 ligands to *BCL*-2, *HRAS1*, *HRAS2*, *VEGF* G4-forming sequences were examined by fluorescence-based microtiter plate assay. The differences in fluorescence intensities between methylated and unmethylated G4 DNAs were statistically significant. In addition to fluorescence detection, the binding of G4 ligand to DNA was detected by chemiluminescence. A significant difference was also detected in chemiluminescence intensity between methylated and unmethylated DNA. This is the first study on the detection of CpG methylation in G4 structures, focusing on structural changes using G4 ligands.

## 1. Introduction

DNA methylation is one of the main epigenetic modifications, and the most abundant DNA methylation form in the genome involves adding a methyl group to the fifth carbon of cytosine (C), forming 5-methylcytosine (5 mC) [1]. DNA methylation predominantly occurs in CpG dinucleotides (CpG), and high densities of CpG are found in CpG islands (CGIs). In the human genome, approximately 70% of gene promoters are associated with a CGI [2]. The DNA methylation level of certain genomic sites represents a potential biomarker of several diseases [3,4]. Commonly used methods for genome-wide DNA methylation analysis are based on bisulfite conversion [5], affinity enrichment by methyl CpG-binding domain (MBD) proteins [6,7], or antibodies specific for 5 mC [8]. However, these methods are time-consuming, complicated, and require expensive reagents. We have previously detected DNA methylation using MBD-based methylated DNA precipitation [9,10]. These systems are sensitive for DNA methylation detection but require a simple detection flow. It is important to address these problems to commercialize methylated DNA analysis for early and pre-symptomatic disease diagnosis [11,12].

In this study, we focused on guanine quadruplex (G4) structures to analyze DNA methylation. G4s are non-canonical steric structures formed by guanine-rich nucleic acids [13,14]. The four guanines form a planar structural unit by Hoogsteen binding, termed a G-tetrad. G-tetrads are held together by central monovalent cations (K^+^ and Na^+^), and G4 is formed by stacking G-tetrads [15]. We have identified G4-forming oligonucleotides in the CGI region by CGI microarray analysis [16]. Recently, it has been reported that roughly 80% of G4 peaks obtained by G4-ChIP-Seq [17] overlap a CGI, and G4 structure presence and stability affect DNA methylation at CGIs [18,19].

**Figure 1:**
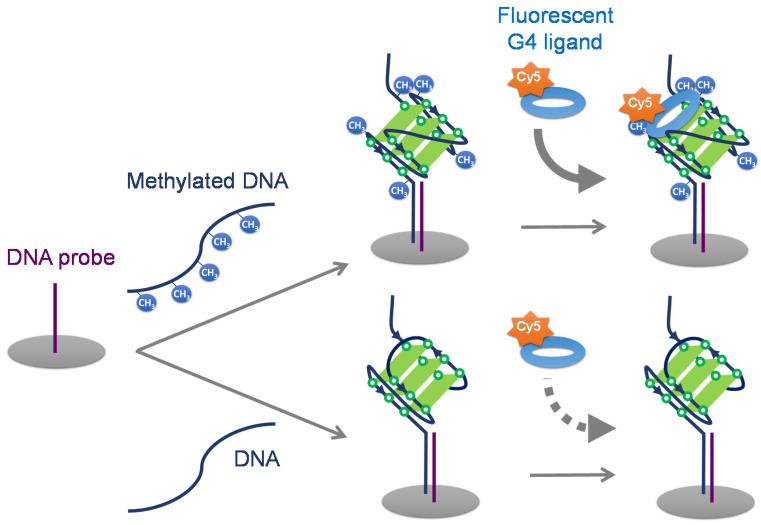
Schematic representation of DNA methylation detection in G4-forming sequences using G4 ligands.

CpG methylation in G4-forming oligonucleotides affects G4 structure stability [20] and topology [21]. We have previously focused on the differences in G4 structure stability and demonstrated that the initial elongation efficiency of PCR decreases with increasing CpG methylation in G4-forming sequences [22]. G4 topology is dependent on the ion type and nucleotide sequence [23,24,25]. We have revealed that cytosine methylation in G4 structure also affects G4 topology using circular dichroism (CD) spectrum measurements [21]. This indicates that DNA methylation can be detected from the differences in G4 structure topology. Then, we considered that DNA methylation could be detected through the differences in G4 topologies using G4 ligands. G4 ligands are small molecules that are exploited to specifically target G4 structures. L1H1-7OTD (7OTD) and L2H2-6OTD (6OTD) are G4 ligands that strongly and selectively bind with G4 DNAs [26,27]. The fluorescent G4 ligand L1Cy5-7OTD effectively detects G4 structures in CGI microarrays [16]. In addition, another cyclic hexaoxazole bearing a vinylnaphthalene group (OTD-VN) fluoresces when bound to G4 DNAs [28]. The restriction of the free rotation of the bond between the oxazole and naphthyl groups upon interaction with G4 DNAs increases the planarity of OTD-VN, resulting in fluorescence. G4 DNAs can be detected simply by mixing them with OTD-VN without removing the free ligand. We hypothesized that these fluorescent ligands would be suitable for detecting DNA methylation. In this study, we aimed to develop a method for detecting DNA methylation using G4 ligands by focusing on the altered G4 topology due to methylation (Figure 1).

## 2. Results and Discussion

### 2.1. Detection of G4 DNA Methylation Using Fluorescent G4 Ligands

**Figure 2:**
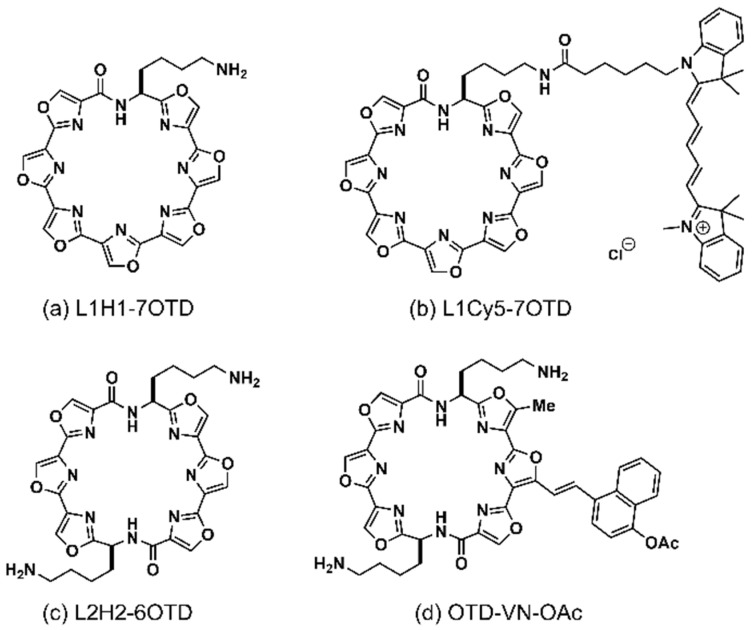
Chemical structures of G4 ligands. (**a**) L1H1-7OTD, (**b**) L1Cy5-7OTD, (**c**) L2H2-6OTD, and (**d**) OTD-VN derivative OTD-VN-OAc.

We examined the binding abilities of fluorescent G4 ligands to methylated and unmethylated G4 DNAs by fluorescence detection. In this study, we used four G-rich DNA sequences as the test sequences, *BCL-2* G4, *HRAS2* G4, *VEGF* G4, and *HRAS1* G4, which contain four, five, three, and five CpG sites, respectively (Table S1). We used sequences in which all CpG sites were methylated as the methylated DNA. These G-rich sequences have been identified in the promoter regions of genes involved in tumorigenesis and fold into G4 structures [20,29,30]. The methylation of these CpG sites in endogenous DNA and the correlation between methylation of these sites and tumorigenesis has not yet been reported, but the methylation of them is associated with cancer. Therefore, we considered these G4 sequences suitable as biomarker models for pre-symptomatic diagnosis. As fluorescent G4 ligands, L1Cy5-7OTD (Figure 2b) and OTD-VN-OAc (Figure 2d), based on 7OTD and 6OTD, respectively, were used. OTD-VN-OAc, a derivative of OTD-VN, is a G4-selective turn-on type ligand and fluoresces by binding to G4-forming DNAs [28].

**Figure 3:**
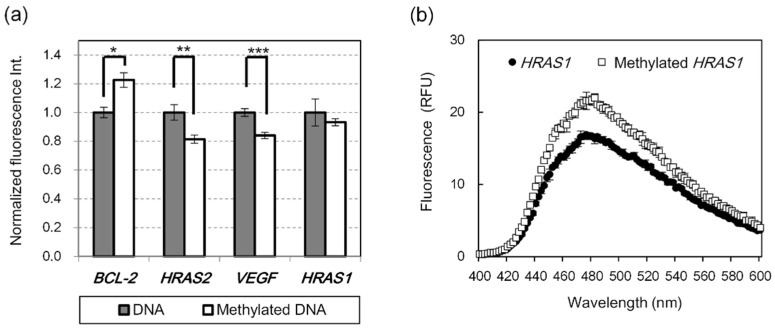
Binding ability of (**a**) L1Cy5-7OTD to *BCL-2* G4, *HRAS2* G4, *VEGF* G4, and *HRAS1* G4 DNAs and (**b**) OTD-VN-OAc to *HRAS1* G4 DNA. (**a**) Bar graphs represent mean values of the normalized fluorescence intensities (normalized by the signal of unmethylated DNA for each DNA). (**b**) The plots denote the mean fluorescence intensities at each wavelength. The error bars represent the standard deviation of the experimental data (*n* = 3 or 4). Statistical analyses were performed using *t*-test. The significance of differences of observed values are indicated as follows: * *p* < 0.01, ** *p* < 0.001, *** *p* < 0.0001.

First, we attempted to detect G4 DNA methylation using L1Cy5-7OTD. L1Cy5-7OTD comprises a Cy5 fluorophore linked to 7OTD; thus, the binding of 7OTD to G4 DNA can be detected by Cy5 fluorescence. The methylated and unmethylated G4 DNAs were biotinylated at the 5′-end and immobilized on a streptavidin-coated plate. After L1Cy5-7OTD was added to the DNA-immobilized plate, the plate was washed, and the fluorescence intensities of Cy5 were observed. The fluorescence intensity of methylated *BCL-2* G4 DNA was significantly higher than that of unmethylated *BCL-2* G4 DNA (Figure 3a). When the fluorescence intensity and the methylation level are linearly related, the difference in fluorescence intensity between unmethylated *BCL-2* G4 (0% methylation) and methylated *BCL-2* G4 (100% methylation) is sufficient to distinguish more than 50% difference in methylation, considering the standard deviation. However, the fluorescence intensity of unmethylated *HRAS2* G4 and *VEGF* G4 DNAs were about 1.2-times stronger than those of methylated DNAs (Figure 3a). On the contrary, the difference in fluorescence intensity between methylated and unmethylated *HRAS1* G4 was insignificant (Figure 3a). To con-firm the conformations of these methylated and unmethylated G4 DNAs, the CD spectra of the DNAs were measured. The CD spectrum of *BCL-2* G4 showed two positive peaks at 265 and 295 nm (Figure S1a), *HRAS1* G4 showed a positive peak at 290 nm (Figure S1b), and *HRAS2* G4 and *VEGF* G4 showed a positive peak at around 260 nm (Figure S1c,d). These results indicate that *HRAS2* G4 and *VEGF* G4 might fold into parallel-type G4 structure, *HRAS1* G4 into anti-parallel type, and *BCL-2* G4 might have a parallel–antiparallel mixed-type G4 structure. The topology of these G4 structures is consistent with previous reports, and some of them have been confirmed by other structure determination methods [31,32]. As reported in the previous report [20,21], the difference between methylated and unmethylated DNA was clearly observed by CD spectrum measurement of *BCL-2* G4 and *HRAS1* G4 (Figure S1a,b). This result suggests that the methylation of the cytosine in G4 DNA induces the conformational change of G4 structure. Although the mechanism of the G4 topology change due to methylation has been unclear, the presence of a bulky methyl group at the C-5 position of the cytosine, which is in the loop or adjacent region of G4 structures, may affect G4 topologies. Furthermore, methylation might add CH-π interactions in G4 structure. It was confirmed that CpG methylation affected the CD spectra of *BCL-2* G4 and *HRAS1* G4, even in the presence of L1Cy5-7OTD (Figure S1e,f). The binding assays using L1Cy5-7OTD showed significant difference in fluorescence intensities between methylated and unmethylated DNA with *BCL-2* G4 but not with *HRAS1* G4. Furthermore, CpG methylation of *HRAS2* G4 and *VEGF* G4 could be detected by L1Cy5-7OTD, even though the differences in the CD spectra of them due to methylation were not so large (Figure S1c,d,g,h). The interaction between fluorescent 7OTD and G4 DNA may be sensitive to changes in parallel-type G4, but not in antiparallel-type G4. For this reason, it might be difficult to observe the difference in fluorescence intensity between methylated and unmethylated *HRAS1* G4 in the binding assay with L1Cy5-7OTD.

OTD-VN-OAc, which has the same framework as 6OTD, is a turn-on type G4 ligand [28]. The restriction of the free rotation of the bond between the oxazole and naphthyl groups upon interaction with G4 DNAs is thought to increase the planarity of OTD-VN derivatives and result in fluorescence [28]. This property enables simple G4 DNA detection, which does not require bound/free separation. Therefore, we attempted to detect DNA methylation using the OTD-VN-OAc. *BCL-2* G4 and *HRAS1* G4 were used as DNA models because the difference between methylated and unmethylated DNA was clearly observed by CD spectrum measurement of these DNAs (Figure S1a,b). Methylated or unmethylated *BCL-2* G4 and *HRAS1* G4 DNAs were incubated with OTD-VN-OAc, and the fluorescence intensity was measured. The fluorescence spectra of OTD-VN-OAc mixed with *HRAS1* G4 are shown in Figure 3b. The fluorescence intensity of methylated *HRAS1* G4 was higher than that of unmethylated *HRAS1* G4. We found a significant difference in fluorescence intensity at λ_em_ 480 nm (the wavelength of maximum fluorescence intensity) between methylated and unmethylated *HRAS1* G4. This difference in fluorescence intensity distinguishes between the differences in DNA methylation rates above 30%. However, no significant difference was found in *BCL-2* G4 (data not shown). For both G4 DNAs, the CD spectra of methylated and unmethylated G4 were slightly different in the presence of OTD-VN-OAc (Figure S1i,j). However, the binding ability of OTD-VN-OAc to G4 DNA was affected by CpG methylation only in *HRAS1* G4. The methyl groups added to the loop region of *HRAS1* G4 may not only affect the G4 structure, but also directly affect the binding of OTD-VN-OAc to *HRAS1* G4.

In this study, we focused on the conformational changes in G4 structure due to CpG methylation and proposed a detection method for CpG methylation in G4 forming sequences using G4 ligands. We have shown that DNA methylation can be detected with a very simple assay using two types of G4 ligands. In particular, using the turn-on type ligand OTD-VN-OAc, DNA methylation was detected simply by mixing DNA and ligand without removing the free ligand. However, this method is limited to the sequences that can form G4 structures, and the relationship between the signal intensity of methylated and unmethylated DNA depends on the sequence of the target DNA and the G4 ligand used. The detection resolution of the methylation level using this method is not as good as that of bisulfite conversion-based methods. However, it is sufficient for analyzing a significant difference in methylated DNA level. The correlation between cancer and DNA methylation is being actively studied [33], and CpG sites with significantly different methylation levels have been reported between samples from cancer patients and control subjects. The methylation level of the O^6^-methylguanine DNA methyltransferase (MGMT) promoter changes by approximately 30% depending on glioma grade [34]. Thus, this technique can be used to distinguish between the DNA methylation levels in different glioma grades. Cell-free DNA (cfDNA) is present in the plasma and serum as double stranded DNA fragments [35,36], and our method has the potential to investigate cfDNA methylation as well as genomic DNA from patients’ specimens. In recent years, it has been reported that cfDNA methylation has great potential as a diagnostic or prognostic biomarker of cancer [37,38,39,40]. For example, overall methylation of particular genes was observed in pancreatic cancer patients but rarely in non-cancer individuals [41]. Furthermore, it has been shown that the cfDNA methylation status of these genes may be able to detect pancreatic cancer during the early stages. We believe that our DNA methylation detection technique may be useful as a diagnostic technique including early diagnosis.

### 2.2. Detection of CpG Methylation in Target DNA Captured by DNA Probe

We attempted to detect CpG methylation in the target DNA containing the G4-forming sequence, which was fixed to the plate via a DNA probe. It is necessary to investigate how to capture the target DNA with a DNA probe, so different ways of capture by hybridization using the immobilized probe DNA were investigated. We targeted *BCL-2*-87, which is an 87-mer DNA including *BCL-2* G4 sequence. The sequence of *BCL-2*-87 is found on human chromosome 18 (Table S1). The complementary strands for both ends of *BCL-2*-87 were used as DNA probes (Table S1, 5′-probe and 3′-probe). PolyT-87, where the *BCL-2* G4 portion of *BCL-2*-87 was replaced with thymine, was used as the negative control. Since the T-rich sequence does not form a G4 structure, we thought that polyT-87 was suitable as a negative control.

The binding of the G4 ligand to probe-hybridized *BCL-2*-87 was evaluated by native-PAGE (Figure S2) to prevent the three-dimensional structures from denaturing. In the lanes with L1Cy5-7OTD mixed with 5′-, 3′-, or both-probe-hybridized *BCL-2*-87, Cy5 fluorescence was detected at the same position as the DNA bands. These results revealed that Cy5-7OTD bound to probe-hybridized *BCL-2*-87. However, Cy5 fluorescence was detected at the same position as the DNA band only when polyT-87 were hybridized with a 3′-probe not with a 5′-probe or both probes. This result indicated that L1Cy5-7OTD also bound to the 5′-side 30-mer region of *BCL-2*-87 and polyT-87, the sequence of which potentially forms G4. Based on these results, we hypothesized that a DNA probe designed to expose only the target G4 sequence region as a single-stranded DNA is suitable. Therefore, in subsequent experiments, both 5′- and 3′-probes were used.

The band shift due to CpG methylation in 5′- and 3′-probe-hybridized *BCL-2*-87 was not observed in native-PAGE analysis (Figure S2). To analyze the effects of CpG methylation on the *BCL-2* G4 sequence of *BCL-2*-87, the CD spectra were measured. The CD spectrum of 5′- and 3′-probe-hybridized unmethylated *BCL-2*-87 showed a positive peak at 265 nm and a shoulder around 290 nm, and these peaks were increased and a negative peak at around 240 nm was detected by CpG methylation (Figure S3a). These results indicate that unmethylated and methylated *BCL-2* G4 sequences within probe-hybridized *BCL-2*-87 mainly folded into a parallel-type G4 structure, and CpG methylation promoted parallel-type G4 formation. The difference in CD spectral patterns between methylated and unmethylated *BCL-2*-87 was also observed in the presence of L1H1-7OTD (Figure S3b). This revealed that G4 conformations were different even in the presence of the G4 ligand. Since the CD spectra of *BCL-2* G4 in the presence of L1Cy5-7OTD and L1H1-7OTD were the same (Figure S1e,k), it is considered that the Cy5 fluorophore linked to 7OTD does not affect the interaction between *BCL-2*-87 and 7OTD.

**Figure 4:**
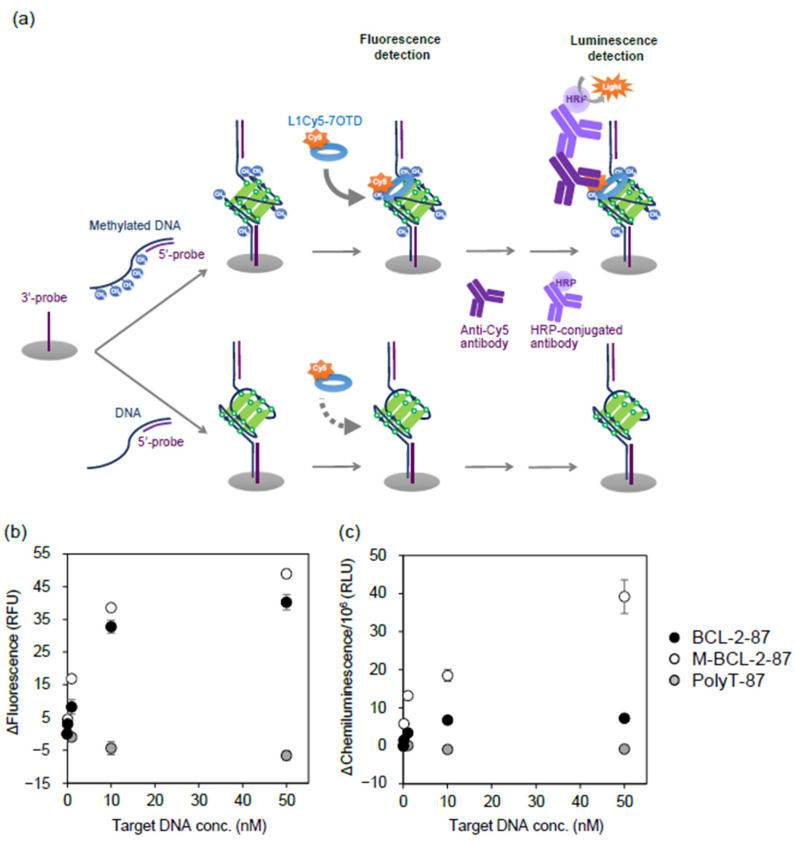
Binding assay of L1Cy5-7OTD to *BCL-2*-87 and methylated *BCL-2*-87 (M-*BCL-2*-87). (**a**) Target DNAs hybridized with 5′-probe were immobilized on a microplate via 3′-probe, then fluorescence was detected after incubation with L1Cy5-7OTD and the target DNAs, and chemiluminescence was detected after incubation with antibodies. Binding abilities of L1Cy5-7OTD to target DNAs were determined by (**b**) fluorescence intensities of Cy5 and (**c**) chemiluminescence intensities. The plots are the mean values of intensities and the error bars represent the standard deviation of the experimental data (*n* = 3). The values obtained by subtracting the signal values of 3′-probe only (Target DNA 0 nM) were plotted as the (**b**) Δfluorescence and (**c**) Δchemiluminescence values. PolyT-87 was utilized as a negative control.

Then, using L1Cy5-7OTD, we attempted to detect the methylation of *BCL-2*-87 immobilized via a DNA probe. The 5′-end biotinylated 3′-probe DNA was immobilized on a streptavidin-coated 96 well plate. After hybridizing *BCL-2*-87, methylated *BCL-2*-87, and PolyT-87 with a 5′-probe, they were immobilized by hybridizing with a 3′-probe (Figure 4a). After adding L1Cy5-7OTD and washing, the fluorescence intensity of Cy5 was measured. The fluorescence intensity increased in a DNA concentration-dependent manner in methylated and unmethylated *BCL-2*-87 but not in PolyT-87 (Figure 4b). At target DNA concentrations above 1 nM, the fluorescence intensities of methylated *BCL-2*-87 were significantly higher than those of unmethylated *BCL-2*-87. Since chemiluminescence does not require excitation light, the background signal can be suppressed, and the signal-to-noise ratio can be improved. Therefore, in addition to fluorescence detection, the binding of L1Cy5-7OTD to DNA was detected by chemiluminescence using anti-Cy5, HRP-conjugated antibodies (Figure 4a). The chemiluminescence intensity showed a DNA concentration-dependent manner in methylated and unmethylated *BCL-2*-87 (Figure 4c). The difference in chemiluminescence intensity between unmethylated and methylated *BCL-2*-87 was greater than the difference in fluorescence intensities (Figure 4b,c), and methylation of 0.1 nM target DNA was detected by chemiluminescence measurement (Figure 4c, Figure S4). The reason for the high-sensitivity detection by chemiluminescence is thought to be that the background signal was suppressed because the chemiluminescence does not require excitation light. Another reason may be the ease of binding of the anti-Cy5 antibody to L1Cy5-7OTD. Since methylated and unmethylated *BCL-2*-87 have different G4 structures, the binding state of L1Cy5-7OTD to them may be different. Anti-Cy5 antibodies might be more likely to bind to L1Cy5-7OTD bound to methylated *BCL-2*-87 than to unmethylated *BCL-2*-87.

We have shown that methylation of the target DNA captured by the DNA probe can be detected by G4 ligand. Since DNA probes can be designed specifically for target DNAs, methylation of multiple target DNAs can be simultaneously analyzed using G4 ligand and microarrays with DNA probes. In fact, many commercial DNA methylation tests use multiple CpG sites as diagnostic biomarkers [33,42]. Our technique may be effective for analyzing multiplex DNA methylation markers such as these.

### 2.3. Detection of CpG Methylation in an MDD Marker Using G4 Ligand

**Figure 5:**
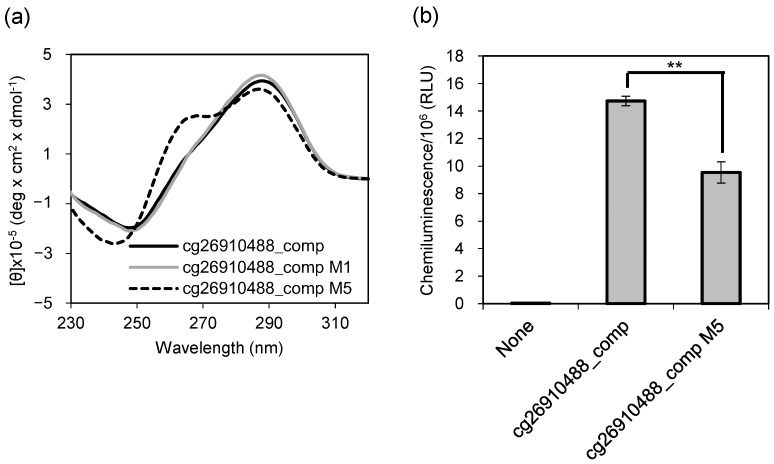
Detection of CpG methylation in a major depressive disorder (MDD) marker. Cg26910488_comp is unmethylated, while cg26910488_comp M1 (including one 5 mC) and cg26910488_comp M5 (including five 5 mCs) are methylated. (**a**) Circular dichroism (CD) spectra of 2 µM cg26910488_comp, cg26910488_comp M1, and cg26910488_comp M5 in 10 mM Tris-HCl, 100 mM KCl, pH 7.4. (**b**) The biding assay of L1Cy5-7OTD to cg26910488_comp and cg26910488_comp M5. Binding abilities of L1Cy5-7OTD to DNAs were determined by chemiluminescence intensities. In the binding assay, as a negative control, a well without immobilized DNA (None) was utilized. Bar graphs represent mean values of the chemiluminescence intensities and the error bars represent the standard deviation (*n* = 3). Statistical analyses were performed using *t*-test. The significance of differences of observed values are indicated as ** *p* < 0.001.

DNA methylation biomarkers have been studied in many diseases, and it has been reported that methylation levels change by nearly 30% depending on glioma grade [34]. As in this case, when the methylation levels of the patients and the healthy controls are significantly different, our method may be useful for disease diagnosis. We then examined whether our method can be applied to the diagnosis of diseases that are clinically important and require pre-symptomatic diagnosis. We have previously conducted genome-wide DNA methylation profiling using Infinium HumanMethylation450 BeadChip, and reported that methylation of 17 CpG sites are useful for distinguishing patients with MDD from non-psychiatric controls [3]. The nucleotide sequences adjacent to these CpG sites have been identified in the human genome. The DNA sequence containing cg26910488, one CpG out of the 17 sites, is C-rich and its complementary strand is G-rich. We thought this complementary strand would form G4. Since the methylation state of complementary CpG of double-stranded DNA is assumed to be the same, we attempted to detect CpG methylation in the complementary DNA sequence of cg26910488 (Table S2, cg26910488_comp) as a potential MDD marker using a G4 ligand. Two types of methylated DNA, cg26910488_comp M1 (with one methylated CpG, shown in bold in Table S2) and cg26910488_comp M5 (with five methylated CpGs, underlined in Table S2), were prepared. The CD spectra of cg26910488_comp and cg26910488_comp M1 showed a positive peak at approximately 289 nm and a shoulder peak at approximately 265 nm (Figure 5a, black and gray solid lines). These results indicated that cg26910488_comp and cg26910488_comp M1 formed a structure similar to that of the hybrid-type G4, and a CpG methylation within cg26910488_comp sequence did not cause enough conformational changes to alter the CD spectrum. In contrast, the CD spectrum of cg26910488_comp M5 was different from that of cg26910488_comp, with two positive peaks at 265 nm and 289 nm and a negative peak at 244 nm (Figure 5a, dotted line), revealing that the methylation of five CpG sites induced a parallel-type G4 structure.

We observed that the CD spectra of cg26910488_comp and cg26910488_comp M5 were different. Then, we attempted to detect the methylation of them using the G4 lig-and. To confirm that CpG methylation of the MDD marker sequence can be detected, the 5′-end biotin-labeled cg26910488_comp and cg26910488_comp M5 were directly immobilized on a streptavidin-coated plate. After adding L1Cy5-7OTD to the DNA-immobilized plate, they were incubated with anti-Cy5 and HRP-conjugated antibodies, and the chemiluminescence was detected. The chemiluminescence intensities of cg26910488_comp (completely unmethylated) and cg26910488_comp M5 (fully methylated) were significantly different (Figure 5b). On the contrary, the difference in methylation level of this CpG site between patients with MDD and controls was about 4% [3]. The resolution of this detection system seems insufficient to distinguish several percent differences in methylation rates, and further improvements in measurement accuracy are required. However, it is important to note that we have shown that the principle of CpG methylation detection using G4 ligands can also be applied to disease-related sequences.

In this study, we confirmed that the DNA sequence near CpG, which is a potential MDD biomarker, forms a G4-like structure, and CpG methylation affects its conformation. Furthermore, it was shown that detecting the CpG methylation by the G4 ligand is possible. However, the differences in DNA methylation levels between psychiatric patients and non-psychiatric controls tend to be small [3,43]. To detect these differences, methylation analysis should be highly accurate. Most genome-wide DNA methylation profiling studies reported to date have been performed using the array-based Infinium methylation assay, which cannot comprehensively analyze all CpG sites in the human genome. Recently, the genome-wide DNA methylation patterns have been analyzed using whole genome bisulfite sequencing. If CpG sites where the methylation level is greatly altered in patients with MDD are discovered from these studies in the future, the method developed in this study may be applied for MDD diagnosis.

## 3. Materials and Methods

### 3.1. Oligonucleotides and G4 Ligands

All oligonucleotides employed in this study (Tables S1 and S2) were synthesized by Eurofins Genomics K.K.; Tokyo, Japan or TAKARA BIO INC.; Shiga, Japan. G4 ligands, L1H1-7OTD, L1Cy5-7OTD and OTD-VN-OAc (Figure 2), were synthesized as reported [16,26,27,28].

### 3.2. CD Spectroscopy

The oligonucleotides were diluted in TK buffer (10 mM Tris-HCl, 100 mM KCl; pH 7.4), denatured at 95 °C for 10 min, and then cooled to 25 °C gradually. G4 ligands were added to the DNA samples and incubated for over an hour at room temperature (RT). CD spectra were measured using a J-820 spectropolarimeter (JASCO; Tokyo, Japan) and a quartz cell with 10 mm optical path length (JASCO) at 20 °C. Scanning speed was 100 nm/min, response time was 1 s, and bandwidth was 1 nm.

### 3.3. Binding Analysis of L1Cy5-7OTD to G4 DNAs by Microtiter Plate Assay

The G4-forming DNAs (Table S1; *BCL-2* G4, *HRAS2* G4, *VEGF* G4, and *HRAS1* G4) were biotinylated at the 5′ end and folded by heat treatment in TK buffer, as described above. They were then diluted in TK buffer to 100 nM, 100 µL diluted oligonucleotide sample was added to a streptavidin-coated 96 well plate (Thermo Fisher Scientific; Waltham, MA, USA), and incubated at RT for 1 h. The wells were washed with washing buffer (TK buffer containing 0.05% Tween 20) and incubated with blocking buffer (TK buffer containing 0.05% Tween 20 and 4% skim milk) at RT for 1 h. After washing with the washing buffer, 100 µL L1Cy5-7OTD (500 nM) was added and incubated for 1 h at RT. After washing, the binding ability of L1Cy5-7OTD to G4 DNAs was determined by detecting the fluorescence intensity at λ_ex_ 651 nm and λ_em_ 670 nm using a Varioskan Flash (Thermo Fisher Scientific). The fluorescence intensities of methylated and unmethylated G4 DNAs were compared using the Student’s *t*-test.

### 3.4. Binding Analysis of L1Cy5-7OTD to BCL-2-87 Using a Microtiter Plate Assay

The 5′-biotinylated 3′-probe for *BCL-2*-87 (Table S1) was heated to 95 °C for 10 min and then quenched on ice. The heat-treated 3′-probe was diluted in TK buffer to 100 nM and added to a streptavidin-coated 96 well plate. After incubation at RT for 1 h and washing the wells, blocking buffer was added to the wells and incubated at RT for 1 h. Finally, the wells were washed with washing buffer to prepare a 3′-probe immobilized plate. Methylated or unmethylated *BCL-2*-87 or polyT-87 (Table S1) were mixed with a 5′-probe (Table S1), heated at 95 °C for 10 min, gradually cooled to 25 °C, and added to the 3′-probe-immobilized plate and incubated overnight at RT. After washing the wells with washing buffer, 100 µL L1Cy5-7OTD (500 nM) was added and incubated for 1 h at RT. After washing, the fluorescence intensity of Cy5 was detected (λ_ex_ 651 nm and λ_em_ 671 nm). Next, 100 µL 5000-fold diluted anti-Cy5 antibody (mouse monoclonal) (Sigma-Aldrich; St. Louis, MO, USA) was added and incubated for 1 h at RT. After washing the wells, 100 µL 5000-fold diluted anti-mouse IgG horseradish peroxidase (HRP)-conjugate (Promega; Madison, WI, USA) was added. After incubation for 1 h at RT, the wells were washed with the washing buffer and 100 µL BM Chemiluminescent ELISA Substrate (POD) (Roche Diagnostics GmbH; Mannheim, Germany) was added. The chemiluminescence intensities were measured at RT using a Varioskan Flash.

### 3.5. Binding Analysis of L1Cy5-7OTD to Major Depressive Disorder (MDD) Marker DNA by Microtiter Plate Assay

The 5′-biotinylated MDD marker model oligonucleotides (Table S2) were folded by heat treatment in TK buffer as described above. The oligonucleotides were diluted in TK buffer to 100 nM and added to a streptavidin-coated 96 well plate. After washing and blocking the wells, 100 µL L1Cy5-7OTD (500 nM) was added and incubated for 1 h at RT. After incubation with anti-Cy5 antibody and anti-mouse IgG HRP-conjugate, chemiluminescence was detected as described above.

### 3.6. Binding Analysis of OTD-VN-OAc to G4 DNAs

The folded *BCL-2* G4 and *HRAS1* G4 (Table S1, final concentration (f.c.) 5 µM) were mixed with OTD-VN-OAc (f.c. 25 µM) in TK buffer. After overnight incubation at RT, the fluorescence intensity at λ_ex_ 370 nm and λ_em_ 400–600 nm was detected using a Varioskan Flash.

## 4. Conclusions

In this study, we focused on the conformational changes in G4 structure due to CpG methylation and proposed a detection method for CpG methylation in G4-forming sequences using G4 ligands. Although this method is limited to the sequences that can form G4 structures, it detects DNA methylation more rapidly and easily than existing bisulfite conversion- or affinity enrichment-based detection methods. The binding assay of the G4 ligand to the target DNA detected methylated DNA at nanomolar concentrations. This result shows that this G4 ligand-based DNA methylation detection method can be highly sensitive. Furthermore, by using DNA probes, it is possible to capture and analyze specific G4 regions of target DNA such as genomic DNA and cfDNA. Genomic DNA extracted from leukocytes in peripheral blood is usually used to analyze genomic DNA methylation as a disease biomarker. Since the normal number of leukocytes in blood is between 4000 and 10,000 per microliter [44], the amount of genomic DNA is approximately 10^4^ copies per microliter. When 1 mL of blood is collected, the amount of genomic DNA is 10^7^ copies. Therefore, further improvement in sensitivity is required. However, there are many methods to condense DNA fragments or amplify DNA hybridization signals, which can help improve the sensitivity of this method in the future. The methylation status of cfDNA has potential as an early diagnosis biomarker of disease. Therefore, the proposed DNA methylation detection method would be used as an early diagnostic tool.

## Data Availability

The data presented in this study are openly available in article and Supplementary.

## Supplementary Materials

The following are available online at https://www.mdpi.com/article/10.3390/ijms222313159/s1.
