# Supplementary material for: Detection of CpG Methylation in G-Quadruplex Forming Sequences Using G-Quadruplex Ligands
Source: Int J Mol Sci. 2021 Dec 6;22(23):13159. doi: 10.3390/ijms222313159 (PMC8658440; doi:10.3390/ijms222313159)
Supplement: Supplementary file 1 [file ijms-22-13159-s001.zip › ijms-1463412-supplementary.pdf]

Detection of CpG methylation in G-quadruplex Forming Sequences using G-quadruplex ligands

Hijiri Hasegawa[1], Ikkei Sasaki[2], Kaori Tsukakoshi[2], Yue Ma[3], Kazuo Nagasawa[2], Shusuke Numata[4], Yuuki Inoue[1], Yeji Kim[1] and Kazunori Ikebukuro[2*]

[1] LG Japan Lab Inc., 4-13-14 Higashi Shinagawa, Shinagawa-ku, Tokyo 140-0002, Japan

[2] Department of Biotechnology and Life Science, Tokyo University of Agriculture and Technology, 2-24-16 Naka-cho, Koganei, Tokyo 184-8588, Japan

[3] Institute of Global Innovation Research, Tokyo University of Agriculture and Technology, 2-24-16 Naka-cho, Koganei, Tokyo 184-8588, Japan

[4] Department of Psychiatry, Graduate School of Biomedical Sciences, Tokushima University, 3-8-15 Kuramoto-cho, Tokushima 770-8503, Japan

* Corresponding Author

E-mail: ikebu@cc.tuat.ac.jp

Table of Contents

Materials and Methods

Native-polyacrylamide gel electrophoresis analysis.

Supporting Figures

Table S1. DNA sequences used in this study.

Figure S1. Circular dichroism spectra of unmethylated and methylated G-quadruplex DNAs.

Figure S2. Native-polyacrylamide gel electrophoresis of DNA/L1Cy5-7OTD complex.

Figure S3. Circular dichroism spectra of *BCL-2*-87 and methylated *BCL-2*-87.

Figure S4. Binding assay of L1Cy5-7OTD to *BCL-2*-87 and methylated *BCL-2*-87.

Table S2. DNA sequence of major depressive disorder marker model DNA.

MATERIALS AND METHODS

Native-polyacrylamide gel electrophoresis (Native-PAGE) analysis. Methylated or unmethylated *BCL-2*-87 or polyT-87 were mixed with a 5′-probe, 3′-probe, or both (Table S1). The heat-treated oligonucleotide samples (final concentration (f.c.) 500 nM) were mixed with L1Cy5-7OTD (f.c. 2.5 µM) in TK buffer (10 mM Tris-HCl, 100 mM KCl; pH 7.4). After incubation at RT for 1 h, the mixtures were loaded on 15% polyacrylamide gel, which was separated at a constant current of 20 mA in TBE buffer (89 mM Tris-HCl, 89 mM H_3BO_4, 2 mM EDTA; pH 8.3). L1Cy5-7OTD was visualized by measuring Cy5 fluorescence using Typhoon 8600 (GE Healthcare; Little Chalfont, UK). The oligonucleotides were stained with SYBR Gold Nucleic Acid Gel Stain (Thermo Fisher Scientific) and scanned using a Gel Doc EZ Imager (Bio-Rad Laboratories; Hercules, CA, USA).

Table S1. DNA sequences used in this study. The methylated CpG sites are underlined.

Name	DNA Sequence (5′→3′)
BCL-2 G4	CGGGCGCGGGAGGAAGGGGGCGGGAGC
HRAS2 G4	CGGGGCGGGGCGGGGGCGGGGGCG
VEGF G4	GGGGCGGGCCGGGGGCGGGG
HRAS1 G4	TCGGGTTGCGGGCGCAGGGCACGGGCG
BCL-2-87	GCGGGCGGGCGGCAGGCGGCGCGGAGGGGCGGGCGCGGGAGGAAGGGGGCGGGAGCGGGGCTGTGGTGCCTGTCCTCTTACTTCAT
PolyT-87	GCGGGCGGGCGGCAGGCGGCGCGGAGGGGTTTTTTTTTTTTTTTTTTTTTTTTTTTTGGGGCTGTGGTGCCTGTCCTCTTACTTCAT
5´-probe	CCCCTCCGCGCCGCCTGCCCGCCCGCCCGC
3´-probe	ATGAAGTAAGAGGACAGGCACCACAGCCCC

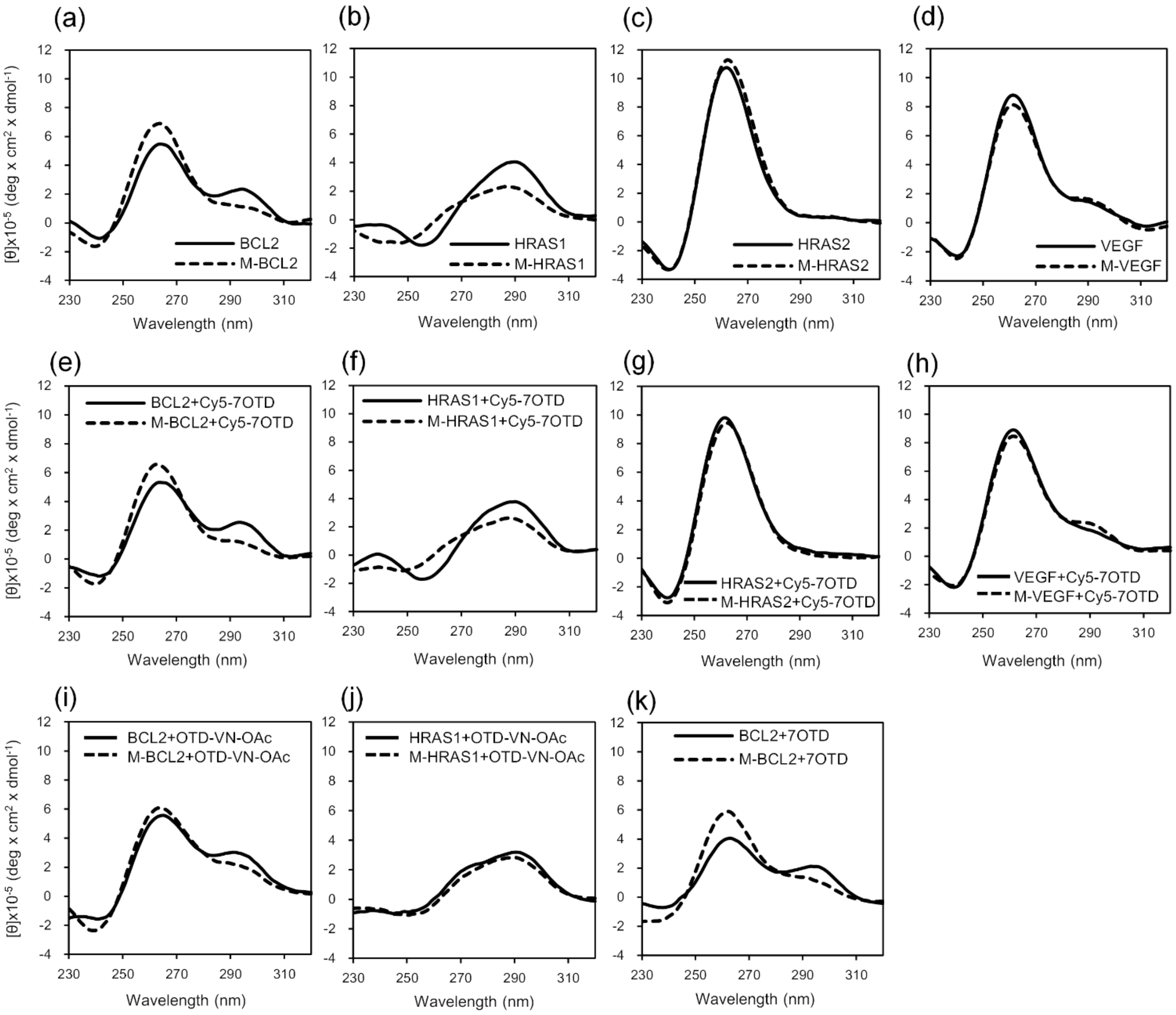

Figure S1. Circular dichroism (CD) spectra of unmethylated (solid line) and methylated (dot line) (a) *BCL-2* G4, (b) *HRAS1* G4, (c) *HRAS2* G4, and (d) *VEGF G4* in 10 mM Tris-HCl, 100 mM KCl, pH 7.4; (e) *BCL-2* G4, (f) *HRAS1* G4, (g) *HRAS2* G4 and (h) *VEGF* G4 in the presence of L1Cy5-7OTD; and (i) *BCL-2* G4, (j) *HRAS1* G4 in the presence of OTD-VN-OAc; and (k) *BCL-2* G4 in the presence of L1H1-7OTD. DNA concentration: 1 µM, L1Cy5-7OTD, OTD-VN-OAc and L1H1-7OTD concentrations: 5 µM.

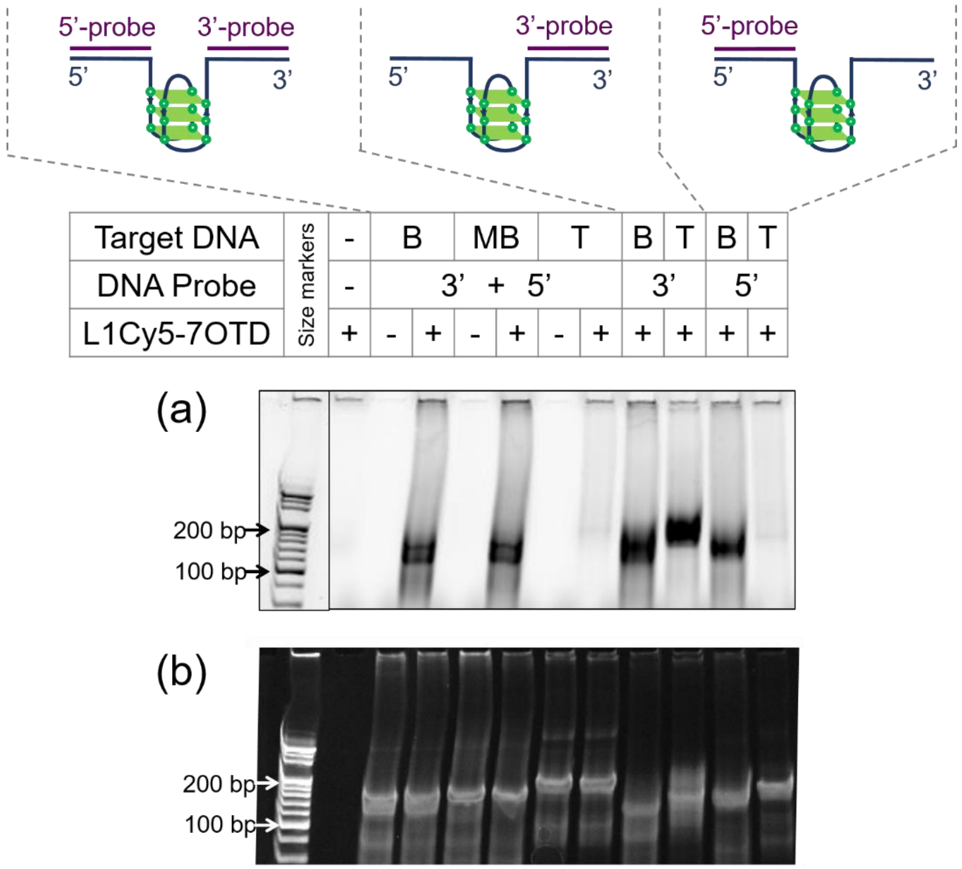

Figure S2. Native-polyacrylamide gel electrophoresis (Native-PAGE) of DNA/L1Cy5-7OTD complex. *BCL-2*-87 (B), methylated *BCL-2*-87 (MB), polyT-87 (T) were hybridized with 3′-probe (3′), 5′-probe (5′), or both probe (3′+5′), and these DNA samples were mixed with L1Cy5-7OTD. (a) Cy5 of L1Cy5-7OTD was detected. (b) DNAs were stained with SYBR Gold and detected. 20 bp ladder pre-mixed with DNA binding fluorescent dye was used as size markers.

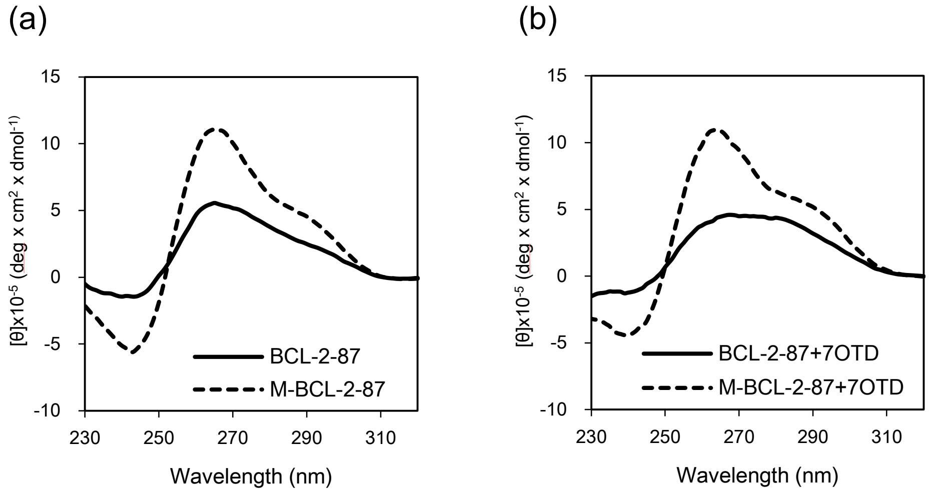

Figure S3. Circular dichroism (CD) spectra of 1 μM *BCL-2*-87 and methylated *BCL-2*-87 (M-*BCL-2*-87) in (a) 10 mM Tris-HCl, 100 mM KCl, pH 7.4 and (b) in the presence of 5 μM L1H1-7OTD. *BCL-2*-87 and M-*BCL-2*-87 were hybridized with 3′-probe and 5′-probe. The CD spectra of the *BCL-2* G4 sequence in *BCL-2*-87 and M-*BCL-2*-87 were determined by subtracting the CD spectrum of a mixed sample consisting of 3′ and 5′ probes and their complementary strands.

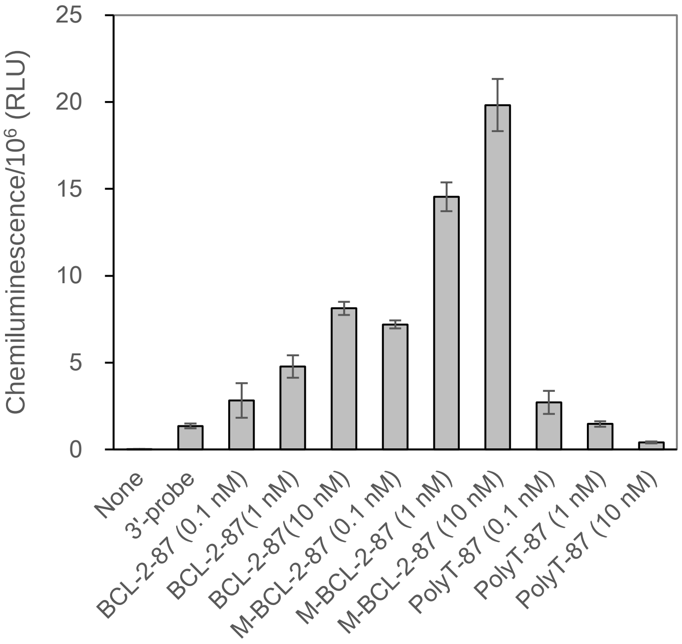

Figure S4. Binding assay of L1Cy5-7OTD to *BCL-2*-87 and methylated *BCL-2*-87 (M-*BCL-2*-87). Binding abilities of L1Cy5-7OTD to target DNAs were determined by chemiluminescence intensities. Target DNAs hybridized with 5′-probe were immobilized on a microplate via 3′-probe, then chemiluminescence was detected after incubation with L1Cy5-7OTD and antibodies. PolyT-87 was used as a negative control.

Table S2. DNA sequence of major depressive disorder (MDD) marker model DNA. The methylated CpG sites are underlined. CpG site reported as MDD marker is shown in bold.

Name	DNA Sequence (5′→3′)
cg26910488_comp	GGGGAGGCCGTGAGCTCGGAAAGGCACCG**CG**GGGCCGGCATTAGGGC
